# A prospective multicentre study testing the diagnostic accuracy of an automated cough sound centred analytic system for the identification of common respiratory disorders in children

**DOI:** 10.1186/s12931-019-1046-6

**Published:** 2019-06-06

**Authors:** Paul Porter, Udantha Abeyratne, Vinayak Swarnkar, Jamie Tan, Ti-wan Ng, Joanna M. Brisbane, Deirdre Speldewinde, Jennifer Choveaux, Roneel Sharan, Keegan Kosasih, Phillip Della

**Affiliations:** 1 Curtin University, School of Nursing, Midwifery and Paramedicine, Kent Street, Bentley, Western Australia 6102 Australia; 2 Department of Paediatrics, Joondalup Health Campus, Suite 204, Cnr Grand Blvd and Shenton Ave, Joondalup, Western Australia 6027 Australia; 3 Department of Emergency Medicine, Perth Children’s Hospital, 15 Hospital Ave, Nedlands, Western Australia 6009 Australia; 4 The University of Queensland, School of Information Technology and Electrical Engineering, Sir Fred Schonell Drive, St Lucia, Brisbane, QLD Australia; 5 Joondalup Health Campus, Cnr Grand Blvd and Shenton Ave, Joondalup, Western Australia 6027 Australia

**Keywords:** Cough, Childhood, Respiratory, Diagnosis, Algorithm, Croup, Pneumonia, Asthma, Bronchiolitis

## Abstract

**Background:**

The differential diagnosis of paediatric respiratory conditions is difficult and suboptimal. Existing diagnostic algorithms are associated with significant error rates, resulting in misdiagnoses, inappropriate use of antibiotics and unacceptable morbidity and mortality. Recent advances in acoustic engineering and artificial intelligence have shown promise in the identification of respiratory conditions based on sound analysis, reducing dependence on diagnostic support services and clinical expertise. We present the results of a diagnostic accuracy study for paediatric respiratory disease using an automated cough-sound analyser.

**Methods:**

We recorded cough sounds in typical clinical environments and the first five coughs were used in analyses. Analyses were performed using cough data and up to five-symptom input derived from patient/parent-reported history. Comparison was made between the automated cough analyser diagnoses and consensus clinical diagnoses reached by a panel of paediatricians after review of hospital charts and all available investigations.

**Results:**

A total of 585 subjects aged 29 days to 12 years were included for analysis. The Positive Percent and Negative Percent Agreement values between the automated analyser and the clinical reference were as follows: asthma (97, 91%); pneumonia (87, 85%); lower respiratory tract disease (83, 82%); croup (85, 82%); bronchiolitis (84, 81%). Conclusion: The results indicate that this technology has a role as a high-level diagnostic aid in the assessment of common childhood respiratory disorders.

**Trial registration:**

Australian and New Zealand Clinical Trial Registry (retrospective) - ACTRN12618001521213: 11.09.2018.

## Background

In paediatrics, respiratory disorders represent the second most common reason for attendance at Emergency Departments (ED) [1, 2] and are a significant global disease burden [3]. Common conditions in childhood include croup, upper respiratory tract infections (URTI), and lower respiratory tract diseases (LRTDs) such as asthma/reactive airway disease (RAD), bronchiolitis, pneumonitis and pneumonia [2, 4]. Lower respiratory tract infections are a significant cause of mortality in children aged under 5 years and a leading cause of disability-adjusted life years lost worldwide [5–7]. Asthma represents the leading cause of non-fatal disease burden in Australian children under age 14 years [8, 9].

The differential diagnosis of respiratory disorders can be challenging even for experienced clinicians with access to diagnostic support services. Respiratory diagnosis may require multiple assessment modalities including clinical and auscultatory examinations, medical imaging, bronchodilator-response testing, spirometry and body fluid analyses. The accurate identification of airway sounds during auscultation is dependent on clinical training and experience [10, 11]. The ability to select and undertake appropriate testing may be restricted in many settings including in community health care and remote areas because of limitations with access to clinical expertise and tests. In hospitals with access to imaging and laboratory services, diagnostic support testing requires resources in terms of clinical staffing, time and monetary costs. Moreover, studies have consistently reported difficulties with inter-rater reliability in radiographic interpretation [12–14]. Diagnostic delays and errors can result in suboptimal therapy with negative implications for morbidity, mortality [15], and antibiotic stewardship [16].

We have previously described a method to diagnose pneumonia based on the automated analysis of cough sounds [17]. With pilot studies demonstrating a sensitivity of 94% and specificity 88% for differentiating pneumonia from no disease, this method has been shown to outperform the World Health Organization (WHO) clinical algorithm for pneumonia diagnosis in resource-poor regions [18]. Subsequently, we have described similar technology for the diagnosis of croup, the most common cause of upper airway obstruction in children between 6 months and 6 years; [19, 20] and reported on the tool’s ability to predict spirometry readings in adults with chronic lung disease [21]. In these studies, automated cough sound analysis required minimal operator training, and was able to provide robust diagnostic accuracies for the specified conditions without the need for clinical auscultation or diagnostic support testing.

We propose that the lungs are connected to the atmosphere via an unimpeded column of air during a cough. This forced expiratory air column supports a greater bandwidth than that across the chest wall relied upon in clinical auscultation, and sounds generated inside the lungs propagate through this air column. The pathophysiological changes caused by different respiratory conditions modulate the sound quality. We use methods akin to that in speech recognition technology to analyse cough and associated sound streams. The identification of unique sound signatures characteristic of different conditions led to the development of an algorithm to test cough sounds for the presence of these signatures [17].

In this study, we used a trained algorithm to analyse a prospective dataset of cough sounds from a cohort with mixed respiratory pathologies. The objective was to compare diagnoses made by the algorithm to those from a clinical adjudication panel (who had access to all medical records and diagnostic support service results) in order to determine positive and negative per cent agreement for a number of respiratory conditions.

The study hypothesis was that automated cough sound analysis is non-inferior to existing standard-of-care clinical diagnosis for identifying URTI, croup and LRTDs (i.e. respiratory disorder below the level of the larynx) including asthma/RAD, bronchiolitis and pneumonia in children.

## Methods

### Study design

This was a prospective, multi-centre study comparing diagnosis of paediatric respiratory illnesses using an automated cough sound analytic algorithm to clinical diagnosis. Investigator teams comprised: (i) data collection and clinical adjudication panel (ii) algorithm development group (iii) index testing team (iv) statistical analyst. The teams were blinded to other team’s work, and clinical diagnoses and index test results were only merged at statistical analysis level.

This study was approved by the Human Research Ethics Committees of Joondalup Health Campus, Princess Margaret Hospital for Children, The University of Queensland and Curtin University. Written informed consent was obtained from all parents/guardians. Children over 5 years were asked for their assent to participate.

### Study sample

Between March 2015 and August 2018, a convenience sample of children aged 29 days to 12 years was recruited into this study at two hospitals in Western Australia; the sole tertiary paediatric hospital in the state and a large suburban general hospital. At each site, enrolment occurred in multiple locations reflecting the intended use of the technology, including emergency departments (ED), inpatient wards and lower acuity ambulatory care units. Inclusion and exclusion criteria are presented in Table 1. Cases were not excluded or stratified by disease severity. All cases presenting to hospital and who met inclusion criteria were eligible.

**Table 1:** Study inclusion and exclusion criteria

Inclusion criteria	
• Age > 29 days and < 12 years	
AND at least one of the following:	
• Rhinorrhoea	
• Cough	
• Wheeze	
• Stridor	
• Increased work of breathing	
• Shortness of Breath	
Exclusion criteria	
• Lack of consent	
• No respiratory disease	
• Mechanical ventilation (invasive, CPAP, or BiPAP) or high-flow nasal cannula	
• Unable to provide at least 5 coughs (Voluntary or spontaneous)	
• Medical contraindication to voluntary cough, including	
○ Severe respiratory distress	
○ History of pneumothorax	
○ Eye, chest, or abdominal surgery past 3 months	
• Too medically unstable to participate in study as per treating clinician	
• Structural airway disease including laryngo/tracheomalacia.	

### Study protocol

Cough sound, demographic and medical data were collected. The study did not intervene with clinical care delivered by the patients’ care providers.

#### Cough recording

The cough recordings and clinical examinations were performed at the same time. Cough audio streams were recorded on iPhone 6 phones (running on instrumentation mode) at a sample rate of 44,100 samples/s and a bit depth of 16 bits per sample held 25-50 cm away from the mouth and with the microphone angled towards the subject at 45 degrees to avoid air hitting the microphone [22]. Recordings were undertaken by a specialist paediatric research nurse in realistic hospital environments where background noises included talking, crying, medical devices, footsteps and doors. Care was taken not to record coughs from other people or television sounds. Between five and ten spontaneous or voluntary coughs were recorded from each child.

Internal work has shown no audio data differences between spontaneous and voluntary coughs.

A proprietary software app developed by ResApp Health was used for the work. Recorded data were analysed off-line on Macintosh computers using proprietary C++ software developed using a machine learning approach. The entire process was automated. An automatic cough detector was developed which identifies cough sounds using Time Delay Neural (TDNN) Network operating and identifying Mel Frequency Cepstral Coefficients (MFCC) from a continuous audio stream [22, 23]. It calculates features from the audio stream to form a feature vector which is then used to classify audio segments as either cough or non-cough by a machine-learning classifier. The classifier has previously been trained from a dataset of manually selected cough and non-cough events. The audio segments are then combined to form completed cough events.

#### Clinical data

Data collected from hospital charts included demographics, medical history, presenting symptoms, vital signs and other clinical (such as auscultatory) findings, response to treatment including bronchodilators, as well as the results of investigations performed.

#### Clinical diagnoses

Table 2 shows the definitions used to arrive at each diagnosis. Definitions were derived by a panel of advisors including physicians from Australia and the USA after consideration of international guidelines [24–26].

**Table 2:** Clinical Diagnosis Definitions

Disease	Required features to reach a clinical diagnosis
Upper respiratory tract disease (URTD)	• Nasal congestion, rhinorrhoea or a sore throat.
Lower respiratory tract disease (LRTD)	• One or more of the following: ○ Wheezing or silent chest (in the setting of obstruction) at the time of recording ○ Any auscultatory findings, including crackles, bronchial breath sounds, or focally decreased breath sounds ○ Increased work of breathing unless purely associated with stridor ○ A productive cough > 5 days ○ New consolidation, infiltrate or pleural effusion on CXR
Asthma/RAD	• Wheeze or silent chest at the time of recording • Responsive to bronchodilators during this illness • Diagnosis is Unsure if: ○ No bronchodilator test^a^ administered ○ Pre-treated with bronchodilators with wheeze resolved at the time of recording
Bronchiolitis	• Age < 24 months • Must have both: o A persistent cough and o Diffuse wheeze that is non-responsive to bronchodilator (if administered) and/or diffuse crackles
Pneumonia (Focal)	At least one feature from both of the following categories: 1. History of: (i) fever in prior 48 h or fever at the time of examination, (ii) cough, (iii) dyspnoea, or (iv) chest pain 2. Either focal^b^ examination findings including crackles, bronchial breath sounds, focal decreased breath sounds; OR A chest radiograph with new consolidation with normal auscultation findings
Croup	• Typical seal-like barking cough on the cough recording.

^a^ Bronchodilator test: administration of Salbutamol MDI via spacer up to 3 times over 1 h at the following doses: 6 puffs for children < 6 yrs., 12 puffs for children > 6 yrs.

^b^ Pneumonia (Focal) implies the absence of generalised findings on auscultation reflecting generalised LRTD such as RAD and bronchiolitis

Each clinical diagnosis was determined by an adjudication panel comprising four consultant paediatric clinicians (median 15 years of specialist practice). Two members reviewed each subject independent of each other, with a third member acting as tie-breaker in the event of non-agreement. The panel arrived at diagnoses after assessment of all available clinical (including radiology) data. Only where a diagnosis of croup was suspected was the panel able to listen to the cough sound files. Clinical diagnoses were concluded before testing of the cough algorithm to ensure blinding was maintained.

There were three outcomes for each disease: “YES”, “NO” or “UNSURE”, where “UNSURE” indicated that the case definition was not entirely met due to lack of information or where symptoms had been significantly altered by treatment before enrolment. These cases were excluded from that disease endpoint. Each subject could be diagnosed as “YES” for more than one disease. For example, LRTD is a broad group which includes all patients with asthma/RAD, bronchiolitis and pneumonia, as well as other conditions meeting the case definitions shown in Table 2.

#### Development of index Test algorithm

The diagnostic algorithm [17, 18, 27] was developed using extraction of mathematical features from cough samples, with selected features used to build a classifier model [17].

In order to refine the algorithm for this study, we used the initial 852 cough sound datasets (collected March 2015-Dec 2016) combined with clinical diagnoses. The method of analysis consisted of automatically picking all cough events from each audio recording, calculating mathematical features such as Mel Cepstral coefficients from coughs, calculating a pre-determined set of signatures and feeding the signatures to a SoftMax neural network trained to diagnose target diseases. The outputs of the SoftMax layer were further processed using probability compositions before a diagnostic decision was reached. These elements were customised and optimised separately for each target disease using the training set; each target disease group had its own signature, SoftMax layer and post-processing logic. An optimal model, using cough sounds only, was designed using a leave-one-out cross-validation procedure. In this method, all available cough sound signals from a single patient were used for testing and cough sound signals from all other patients were used for training the classifier, making the trained model independent of the test patient. This process was repeated for all patients resulting in the number of trained models equal to the number of patients. Each disease model was developed independently using diagnoses for that particular disease only, without consideration of results for other diseases.

A diagnostic algorithm was then developed for the prospective testing phase: Features were selected from a group of up to five parent/guardian-reported symptoms including the presence or absence of (i) fever (ii) rhinorrhoea (iii) audible wheeze (not stridor) (iv) hoarse voice and (v) maximum days of symptoms. The individual clinical features used varied across different disease models. The optimal combination of features was selected using a ROC with due consideration given to achieving a balance of PPA and NPA [17].

The number of cases used for training the algorithm was 50 for croup, 102 for bronchiolitis, 193 for RAD, 72 for pneumonia, 121 for URTI, 522 for LRTD and 123 had no disease.

#### Prospective testing of optimised diagnostic algorithm

Between Dec 2016 – Aug 2018, we recruited children for a prospective diagnostic accuracy trial from the same sites using the same inclusion/exclusion criteria. This group of children (*n* = 585) were independent of the group used to develop the algorithm (*n* = 852). Clinical diagnoses were determined by the clinical adjudication panel and were completed before index test analysis. The analysis of the cough sound files plus symptom data by the algorithm was conducted by an independent researcher unrelated to the clinical sites and the algorithm development team. The algorithm output was compared to the reference clinical diagnosis by an independent statistical team.

For the optimised algorithm as used for this set, automatic segmentation extracted only the 5 five coughs for analysis. For each recording, the algorithm determined diagnoses using cough data plus features picked from a group of five parent/guardian-reported symptoms including the presence or absence of (i) fever (ii) rhinorrhoea (iii) audible wheeze (not stridor) (iv) hoarse voice and (v) maximum days of symptoms (i-iv). No attempt was made to stratify the degree of fever or severity of wheeze. The index test delivered a binary response: “YES” or “NO” for each disease.

### Statistical analysis

Power calculations were derived as follows. Based on expected positive and negative per cent agreement greater than 85% from the training program, to obtain a superiority end-point of 75% (lower bound 95% CI of maximum width ± 0.10) a minimum of 48 cases were required for each disease. Using the prevalence of focal pneumonia (the least prevalent targeted condition) in the training arm of 11%, and assuming a 10% attrition rate, a minimum cohort of 480 were needed.

As clinical diagnoses were considered non-reference standard measures, the primary measures of diagnostic agreement used were Positive Percent Agreement (PPA) and Negative Percent Agreement (NPA). PPA are those clinical diagnosis-positive cases who are also positive for the index test; NPA are those clinical diagnosis-negative cases who are also negative for the index test. 95% confidence intervals around these parameters were calculated using the method of Clopper-Pearson.

## Results

### Study population

**Fig. 1:**
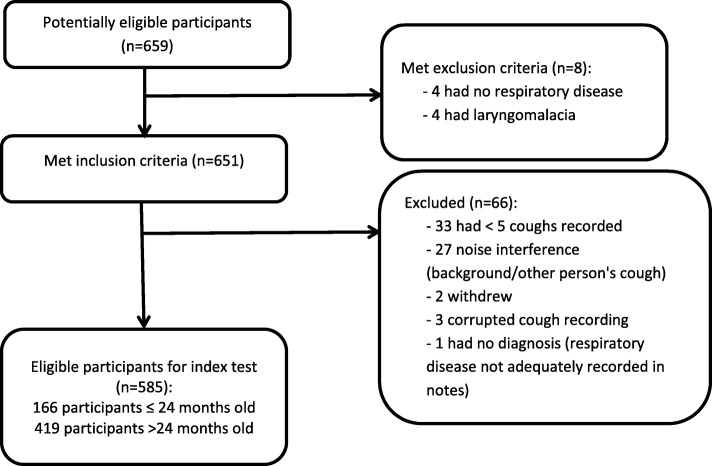
Flow diagram showing enrolment pathway and exclusions

Six hundred fifty-nine subjects were approached to participate of which 585 (88.8%) were used for analysis (Fig. 1). The non-analysed group (*n* = 74) was significantly younger (mean 36 months, SD 29 months) from the analysed group (mean 53 months, SD 37 months). Reflecting technical difficulties with cough collection in the younger cohort, 84% of children ≤24 months were analysable vs 92% of children > 24 months. The groups did not differ in terms of sex. In the analysed group 59% were male, and 28% were ≤ 24 months. 176/585 (30.1%) were ED-only patients, 367/585 (62.7%) were recruited from the inpatient wards, 28/585 (4.8%) were recruited from ambulatory care units and for 14/585 (2.4%) the recruitment site was not recorded.

The number of cases achieving consensus clinical diagnosis and available for index testing is shown in Table 3. A third adjudicator was required to tie-break in 30% of subjects (pneumonia = 6%, asthma/RAD = 18.8%, LRTD = 30%, bronchiolitis = 34%). Of the 419 subjects with a diagnosis of LRTD, 340 had a specific diagnosis of: asthma/RAD (*n* = 149), Bronchiolitis (*n* = 131) or pneumonia (*n* = 60) as per the study definitions. Of these, 1 subject had both asthma/RAD and bronchiolitis, while 3 subjects had both bronchiolitis and pneumonia. The remaining 80 subjects had a LRTD diagnosis not further specified.

**Table 3:** Number of cases per disease group attaining consensus (yes / no) clinical diagnosis

Clinical Diagnosis	Study participants (n)	Subjects without a (yes/no) consensus diagnosis (n)	Subjects used for index testing (n)
LRTD	585	12 (2%)	573
ASTHMA/RAD	585	55 (9.4%)^a^	530
CROUP	585	17 (2.9%)	568
PNEUMONIA	585	16 (2.7%)	569
URTD	585	14 (2.3%)	571
BRONCHIOLITIS	166^b^	9 (5.4%)	157

^a^ RAD: Cases excluded from Index testing due to pre-treatment with bronchodilator leading to wheeze resolution before recording

^b^ Bronchiolitis: 419 cases excluded as > 24 months old

### Diagnostic test results

Table 4 shows PPA and NPA stratified by age for three age groups: 29 days to 12 years, 29 days to 2 years and 2 years to 12 years for each disease.

**Table 4:** Results for Index Test versus Clinical diagnoses per age groups

Clinical Condition	Clinical Diagnosis Positive (n)		Index Test	
	Yes	No	Positive Percent Agreement (95% CI)	Negative Percent Agreement (95% CI)
Subjects 29 days – 12 years				
LRTD	419	154	83% (79–86%)	82% (75–88%)
ASTHMA/RAD	149	381	97% (92–99%)	91% (88–94%)
CROUP	68	500	85% (75–93%)	82% (78–85%)
PNEUMONIA	60	509	87% (75–94%)	85% (82–88%)
URTD	89	482	79% (69–87%)	80% (76–84%)
BRONCHIOLITIS	131	26	84% (77–90%)	81% (61–93%)
Subjects 29 days - 2 years				
LRTD	145	19	88% (82–93%)	74% (49–91%)
ASTHMA/RAD	10	149	80% (44–97%)	97% (93–99%)
CROUP	15	146	80% (52–96%)	79% (72–86%)
PNEUMONIA	6	160	100% (54–100%)	97% (93–99%)
URTD	4	158	50% (7–93%)	87% (80–92%)
BRONCHIOLITIS	131	26	84% (77–90%)	81% (61–93%)
Subjects 2–12 years				
LRTD	274	135	80% (74–84%)	83% (76–89%)
ASTHMA/RAD	139	232	98% (94–100%)	88% (83–91%)
CROUP	53	354	87% (75–95%)	83% (79–87%)
PNEUMONIA	54	349	85% (73–93%)	80% (76–84%)
URTD	85	324	80% (70–88%)	77% (72–82%)

For all children (29 days to 12 years), the asthma/RAD algorithm achieved excellent agreement results (PPA = 97%, NPA = 91%) as did pneumonia (PPA = 87%, NPA = 85%), LRTD (PPA = 83%, NPA = 82%) and croup (PPA = 85%, NPA = 82%). Bronchiolitis achieved PPA of 84% and NPA of 81%. However the paucity of children without respiratory disease under 2 years of age resulted in a wide confidence interval for NPA.

For children aged 2–12 years agreement was also excellent for LRTD (PPA = 80%, NPA = 83%), asthma/RAD (PPA = 98%, NPA = 88%), croup (PPA = 87%, NPA = 83%), pneumonia (PPA = 85%, NPA = 80%) and URTD (PPA = 80%, NPA = 77%).

For children aged 29 days to 2 years, though the results achieved for asthma (PPA = 98%, NPA = 88%), pneumonia (PPA = 85%, NPA = 80%) and URTD (PPA = 80%, NPA = 77%) were high, there were low number of disease positive participants recruited and more data will be required for this age group.

## Discussion

The study results show that the performance of the automated algorithm was not inferior to pre-specified endpoints for diagnosing asthma, croup, pneumonia and lower respiratory tract disease from a group of mixed paediatric respiratory disorders.

The algorithm’s used cough analysis in combination with 5-symptom input obtained from parent/guardian history; without the need for clinical examination or further investigations. The symptoms entered into the algorithm were simple questions that we anticipate most parents can answer irrespective of cultural or educational background. The algorithm’s diagnostic accuracy may be further improved with the additional input of clinical signs such as respiratory rate or chest recessions. This will be examined in a future study.

The results for clinical pneumonia (all ages: PPA 87%, NPA 85%) exceeds other scoring systems including the WHO criteria for clinical pneumonia diagnosis which is based upon clinical signs and symptoms and where sensitivity is prioritised (sensitivity range: 19–96%, specificity range 12–76%) [28]. Whereas earlier work established the system’s ability to isolate pneumonia from any respiratory disease, this study has demonstrated its ability to also differentiate pneumonia and other specified respiratory disorders from a mixed, undifferentiated group.

To address international guidelines aimed at minimising the over-diagnosis of pneumonia, and to establish consistency of diagnosis in the adjudication panel, we defined clinical pneumonia to reflect focal/lobar pathology in the absence of another generalised lower respiratory disease such as RAD, pneumonitis or bronchiolitis. This avoided over-diagnosing pneumonia in generalised LRTDs where radiology was performed and showed abnormalities. The WHO has defined radiological findings to detect bacterial lung disease in post-vaccination surveillance programs [12]. However, a high proportion of children with Respiratory Syncytial Virus positive bronchiolitis were found to have radiographs consistent with the WHO radiological definition for pneumonia (consolidation of a lobe or whole lung); indicating that imaging has reduced predictive value for bacterial disease in settings of high viral activity [29, 30]. Current guidelines recommend against the use of diagnostic imaging in conditions such as bronchiolitis and asthma [26, 31, 32]. Despite this, chest radiographs continue to be inappropriately ordered resulting in unnecessary antibiotic use [33–36].

The algorithm’s performance for RAD was excellent (PPA 97%, NPA 91%) and it is significant that the analyser was able to diagnose RAD at a high-performance level without the need for bronchodilator-response testing. 9.4% of the recruited RAD cases were excluded from analysis because of bronchodilator pre-treatment that resulted in substantially altered symptoms at the time of cough measurement. Study criteria required RAD cases to have wheeze or silent chest as a marker of active disease. Although this excluded partially treated cases where wheeze had resolved, it may be possible for the analyser to detect those in which some obstruction may still be present. Further work is required to investigate this.

We have previously shown that cough analysis has utility in predicting spirometry results in adults [21]. If this is replicated for childhood RAD, the ability to detect disease and measure severity will enhance asthma management programs. The ability to differentiate RAD from pneumonia is particularly important for low-income countries in which guidelines favour pneumonia over RAD diagnosis such that up to 50% of children under 5 years diagnosed with pneumonia could be reclassified as asthma [37]. This has resulted from a focus on not missing infectious disease, as well as the difficulties associated with bronchodilator testing which is time-consuming and requires expertise to interpret. The ability of the cough analyser to differentiate RAD from pneumonia may improve diagnostic accuracy, resulting in more appropriate therapy and improved health resource efficiencies.

In broader terms, the algorithm’s ability to accurately identify LRTDs from pure upper respiratory disease may be useful in triage, telehealth, community and remote area medicine by guiding on when to attend or escalate medical care. The system may be useful as a diagnostic aid in acute care settings, or in places where clinical expertise and diagnostic support services are lacking and have implications for triage-initiated treatment pathways. In consultations where there is limited ability to undertake clinical and auscultatory examinations, the cough analyser may be useful as it can approximate existing standard-of-care clinical diagnoses. The telehealth industry is a rapidly growing health sector with more than 30% of consults per year in the US for respiratory complaints [38]. Significant cost savings may be realised by reducing the need for investigations and improving antibiotic stewardship.

The algorithm can be integrated into smartphones which also serve as the cough sound acquisition device. Advantages of using these devices include they (i) meet acoustic requirements (bandwidth, noise levels, and transduction-sensitivity) needed for respiratory sound recording, (ii) are ubiquitous devices even in the developing world, (iii) possess substantial computing power allowing for analyses with no requirement for connectivity, (iv) enable non-contact assessments ideal for infection control and for use in children or non-compliant subjects, (v) are usable in realistic clinical settings where background noise is a factor. This allows for an all-in-one data acquisition, analysis and decision-making device.

There were a number of study limitations.

A number of sound files were not usable (27; 4.1%). While the technology is appropriate for use in realistic clinical and hospital settings with reasonable background noise, there were practical issues with the unintentional capture of coughs from other people. Awareness of this issue will help ensure that cough-recording captures only the intended subject’s coughs.

In this trial, the algorithm was compared to clinician derived diagnoses. Although the latter is considered a non-reference standard test, it reflects existing best clinical practice. We sought to improve clinical diagnostic accuracy by requiring consensus agreement from an expert panel using strict disease definitions. However 30% of cases required partial adjudication by a third clinician to attain majority decision. In studies with significant variance, it has been shown that there will generally be little incremental benefit to using more than three assessors [39].

The lack of an objective reference standard presents a challenge in the evaluation of new diagnostic tests. In RAD there is subjectivity in the interpretation of bronchodilator response unless objective lung function measurement techniques are used [40]. These techniques are not feasible in young children at the point of care. The differentiation of bronchiolitis and RAD in children under age 2 years is dependent on bronchodilator response. If a bronchodilator test is not administered there may be diagnostic classification errors reflecting age-related variability in airway maturation and bronchodilator responsiveness. The diagnosis of pneumonia relies on appropriate training and resources and is influenced by geographical and community factors [14, 41]. Interpretation of chest radiographs has shown poor inter-rater agreement for pneumonia [13, 42–44].

Care should be taken when assessing the generalisability of these results to different populations. The symptoms entered into the algorithm are reliant on subjective parent reports. Symptom duration (time from the first symptom to medical care attendance) may vary in different populations. It is anticipated that this may be greater in resource-poor and remote areas, and lesser where there is easy access to health care. Telemedicine has the opportunity to reduce these times by improving medical access to isolated communities.

Children under 24 months were not able to cooperate with providing voluntary coughs. This limited the collection of cough data in this group to subjects with spontaneous coughing from LRTD and affected numbers for pure upper airway disease. The NPA for bronchiolitis was affected by the small numbers of children without significant LRTD in the cohort, resulting in a wider 95% CI. The PPA, however, remained robust. There were also smaller numbers with pneumonia in this age group; however, the PPA and NPA were maintained with a wide CI for PPA. Promising PPA and NPA results were demonstrated when examining test agreement in children under 2 years only, however larger studies in children under 24 months are required to determine the utility of the algorithm to younger infants/children in other populations and would be beneficial given bronchiolitis and pneumonia are important diagnostic issues in this age group.

Our study population included both ambulatory care and acutely unwell patients. Although we did not stratify for disease severity, our study population was enrolled predominantly from ED and inpatient wards suggesting higher acuity disease than that encountered in ambulatory care settings. The prevalence and severity of childhood respiratory disorders may vary in different populations.

## Conclusion

We have demonstrated that automated cough analysis delivers good diagnostic accuracy in detecting common childhood respiratory diseases including pneumonia, RAD, croup, bronchiolitis, upper and lower respiratory tract disorders. The technology can be installed onto ubiquitous devices (smartphones), agrees with existing standard-of-care clinical diagnosis and provides a point-of-care diagnosis without the need for clinical examination, supplemental investigations, or bronchodilator testing. It can be used as a diagnostic aid for childhood respiratory disorders. Its use in different settings such as hospitals, ambulatory care, community and telehealth deserves evaluation. Further work will assist in delineating its utility in these areas.

## Abbreviations

CI: Confidence interval
CXR: Chest x-ray
ED: Emergency department
LRTD: Lower respiratory tract disease
NPA: Negative per cent agreement
PPA: Positive per cent agreement
RAD: Reactive airway disease
SD: Standard deviation
URTD: Upper respiratory tract disease
WHO: World Health Organisation
